# A novel real-time PCR assay for specific detection and quantification of *Mycobacterium avium *subsp. *paratuberculosis *in milk with the inherent possibility of differentiation between viable and dead cells

**DOI:** 10.1186/1756-0500-3-251

**Published:** 2010-10-06

**Authors:** Monika Dzieciol, Patrick Volgger, Johannes Khol, Walter Baumgartner, Martin Wagner, Ingeborg Hein

**Affiliations:** 1 Department for Farm Animals and Veterinary Public Health, Institute for Milk Hygiene, Milk Technology and Food Science, University of Veterinary Medicine, Veterinaerplatz 1, A-1210 Vienna, Austria; 2 Department for Farm Animals and Veterinary Public Health, Clinic for Ruminants, University of Veterinary Medicine, Veterinaerplatz 1, A-1210 Vienna, Austria

## Abstract

**Background:**

*Mycobacterium avium *subsp. *paratuberculosis *(MAP) is the etiological agent of paratuberculosis (Johne's disease) in ruminants and is suggested to be one of the etiologic factors in Crohn's disease in humans. Contaminated milk might expose humans to that pathogen. The aim of the present study was to develop a novel real-time PCR assay providing the additional possibility to detect viable *Mycobacterium avium *subsp. *paratuberculosis *(MAP) based on the MAP-specific Mptb52.16 target. The design included an internal amplification control to identify false negative results.

**Findings:**

Inclusivity and exclusivity tested on 10 MAP strains, 22 non-MAP mycobacteria, and 16 raw milk microflora strains achieved 100%. The detection limit in artificially contaminated raw milk was 2.42 × 10^1 ^MAP cells/ml milk. In a survey of naturally contaminated samples obtained from dairy herds with a known history of paratuberculosis, 47.8% pre-milk and 51.9% main milk samples tested positive. Real-time PCR-derived MAP-specific bacterial cell equivalents (bce) ranged from 1 × 10^0 ^to 5.1 × 10^2 ^bce/51 ml; the majority of samples had less than one bce per ml milk. Expression of the chosen target was detected in artificially contaminated raw milk as well as inoculated Dubos broth, thus confirming the real-time PCR assay's potential to detect viable MAP cells.

**Conclusions:**

Concentrating the DNA of a large sample volume in combination with the newly developed real-time PCR assay permitted quantification of low levels of MAP cells in raw milk and pasteurized milk. The selected target - Mptb52.16 - is promising with regard to the detection of viable MAP. Future studies integrating quantitative DNA- and RNA-based data might provide important information for risk assessment concerning the presence of MAP in raw milk and pasteurized milk.

## Background

*Mycobacterium avium *subsp. *paratuberculosis *(MAP) is known as the etiological agent of paratuberculosis (Johne's disease) in ruminants. Symptoms are progressive weight loss and chronic diarrhea associated with granulomatous enteritis. Subclinical infection of cows results in reduced milk production and fertility and signifies a considerable economic loss for the global cattle industry [1]. Crohn's disease is an inflammatory gastrointestinal tract disease in humans, presenting with similar symptoms and pathological changes in the gut as Johne's disease in cattle. Therefore, it was suggested that MAP could be one of the etiologic factors of the disease [2,3]. MAP is possibly passed on to humans through contaminated milk and dairy products although shedding levels appear to be low, especially in subclinical cases (2-8 cfu/50 ml) [4]. However, some data suggest survival during pasteurization [5,6].

The organism is extremely difficult to culture. MAP is one of several slow-growing mycobacteria that require long incubation times. Therefore, samples are frequently lost due to overgrowth of background flora. Decontamination protocols used to control the problem also inhibit the growth of MAP [5]. Thus, alternative DNA-based conventional PCR and real-time PCR detection methods were developed using single- and multi-copy targets such as *hspX*, *F57*, IS*Mav2*, IS*Map02 *and IS900 [6-10]. The multi-copy IS900 target is most commonly used, although IS900-like sequences were reported to be present in other mycobacteria [11-13]. MAP-specific regions being expressed during growth of pure cultures in Dubos broth were identified recently [14]. Based on these findings, the aim of the present study was to develop a MAP-specific real-time PCR assay providing the additional possibility of detecting viable MAP. Pre-treatment of milk samples was optimized in order to analyze DNA isolated from a large volume of milk in a single PCR reaction.

## Methods

### Bacterial strains

The bacterial strains used in this study are listed in Table 1. MAP strains were cultured on selective Herrold's egg yolk medium slants with mycobactin J and amphotericin B, nalidixic acid and vancomycin (HEYA slants; Becton Dickinson, Franklin Lakes, NJ, USA). The slants were incubated at 37°C in horizontal position for 2-6 weeks. The colonies were stained by the Ziehl-Neelsen procedure to confirm the presence of acid-fast bacilli. Non-MAP and raw milk microflora strains were grown according to their individual requirements.

**Table 1:** Bacterial strains used for inclusivity and exclusivity testing of the Mptb52.16 real-time PCR assay

Genus and species	Source
*Mycobacterium avium *subsp. *paratuberculosis*	CIP^a ^103964
*Mycobacterium avium *subsp. *paratuberculosis*	CIP 103967
*Mycobacterium avium *subsp. *paratuberculosis*	CIP 103968
*Mycobacterium avium *subsp. *paratuberculosis*	CIP 103971
*Mycobacterium avium *subsp. *paratuberculosis*	CIP 103972
*Mycobacterium avium *subsp. *paratuberculosis*	CIP 103973
*Mycobacterium avium *subsp. *paratuberculosis*	CIP 103974
*Mycobacterium avium *subsp. *paratuberculosis*	CIP 103975
*Mycobacterium avium *subsp. *paratuberculosis*	CIP 103976
*Mycobacterium avium *subsp. *paratuberculosis*	CIP 103977
*Mycobacterium abscessus*	DSM^b ^44196
*Mycobacterium aurum*	DSM 43999
*Mycobacterium avium subsp.avium*	DSM 44156
*Mycobacterium avium subsp.silvaticum*	DSM 44175
*Mycobacterium chitae*	DSM 44633
*Mycobacterium duvalii*	DSM 44244
*Mycobacterium flavescens*	DSM 43991
*Mycobacterium fortuitum subsp. fortuitum*	DSM 46621
*Mycobacterium gordonae*	DSM 44160
*Mycobacterium intracellulare*	DSM 43223
*Mycobacterium kansasii*	DSM 44162
*Mycobacterium marinum*	DSM 44344
*Mycobacterium neoaurum*	DSM 44074
*Mycobacterium nonchromogenicum*	DSM 44164
*Mycobacterium parafortuitum*	DSM 43528
*Mycobacterium phlei*	DSM 43239
*Mycobacterium scrofulaceum*	DSM 43992
*Mycobacterium shiumoidei*	DSM 44152
*Mycobacterium thermoresistibile*	DSM 44167
*Mycobacterium triviale*	DSM 44153
*Mycobacterium vaccae*	DSM 43292
*Mycobacterium xenopi*	DSM 43995
*Bacillus cereus*	NCTC^c ^7464
*Clostridium bovis*	DSM 20582
*Clostridium pyogenes*	DSM 20630
*Enterococcus faecalis*	ATCC^d ^19433
*Escherichia coli*	NCTC 9001
*Lactococcus lactis*	DSM 20069
*Listeria monocytogenes*	NCTC 11994
*Pseudomonas aeruginosa*	NCTC 10662
*Salmonella typhimurium*	ATCC 14028
*Staphylococcus aureus*	NCTC 1803
*Staphylococcus epidermidis*	ATCC 12228
*Streptococcus agalactiae*	DSM 2134
*Streptococcus dysgalactiae subsp. dysgalactiae*	DSM 20662
*Streptococcus dysgalactiae subsp. equisimilis*	DSM 6176
*Streptococcus thermophilus*	DSM 20617
*Streptococcus uberis*	DSM 20569

Colonies (MAP and non-MAP mycobacteria) suspended in 1 ml of Ringer's solution or overnight broth cultures (raw milk microflora) were subjected to DNA isolation using the NucleoSpin tissue kit (Machery-Nagel, Düren, Germany). The DNA concentration was measured fluorimetrically using a HoeferDyNA Quant200 apparatus (Pharmacia Biotech, San Francisco, USA) and the DNA was diluted to 0.1 ng/μl ddH_2_O.

Notes: ^a ^CIP: Institute Pasteur, ^b ^DSM: German Collection of Microorgansims, ^c ^NCTC: National Collection of Type Cultures, ^d ^ATCC: American Type Culture Collection

Strain CIP103974 was used to artificially contaminate raw milk samples. A colony grown on a HEYA slant was suspended in 1 ml of Ringer's solution (Mayrhofer Pharmazeutika GmbH&Co KG, Leonding, Austria). Cells were declumped by passing the suspension three times through a disposable insulin syringe (Omnican^®^40; B. Braun Melsungen AG, Melsungen, Germany). Debris was removed at 3,000 × g for 1 min. Cells remaining in the supernatant were pelleted at 8,000 × g for 5 min and washed three times in 1 ml Ringer's solution. Again, cells were declumped with a syringe and the cell count in the suspension was determined with a commercial bacterial viability kit (Live/Dead *Bac*Light; Molecular Probes, Willow Creek, OR, USA) and filtration onto 0.22-μm-pore size, 13-mm black polycarbonate filters (Millipore, Billerica, MA, USA).

### DNA isolation of raw milk samples

Separation of MAP from the 98 raw milk samples collected in the Free State of Thuringia in Germany was performed by a method including different detergents, solvents and centrifugation steps [15]. The raw milk samples comprised pre-milk (first streams of milk collected at the beginning of milking) and main milk samples. For each sample, four aliquots of 15 ml of raw milk each were subjected to this protocol. Afterwards, the bacterial pellet was subjected to DNA isolation using the NucleoSpin tissue kit, yielding 100 μl DNA suspension. For each sample, a total of 400 μl DNA suspension (100 μl from each 15 ml aliquot) was concentrated by ethanol precipitation. The DNA pellet was re-suspended in 28 μl ddH_2_O. Thus, the DNA from 60 ml milk was eventually concentrated into a 28-μl volume.

### Comparative DNA and RNA isolation of raw milk samples and inoculated Dubos broth

The bacterial pellet recovered from 15 ml of artificially contaminated raw milk was either subjected to DNA isolation (NucleoSpin tissue kit) or RNA isolation (High Pure RNA Isolation kit; Roche Diagnostics GmbH, Vienna, Austria), or transfered to a 5-ml volume of BBL™ Dubos broth (Becton Dickinson Austria GmbH, Schwechat, Austria) supplemented with 2 mg/ml Mycobactine J (Synbiotics Europe SA, Lyon, France) after decontamination with 0.1% benzalkonium chloride (Sigma-Aldrich Handels GmbH, Vienna, Austria) [16] and incubated at 37°C. Bacteria were recovered at 8,000 × g for 5 min after incubation for 1 or 2 weeks and subjected to DNA and RNA isolation.

### Real-time PCR

The presence of PCR inhibitors in the DNA isolated from milk was tested as previously published [17]. Two-hundred copies of an artificially synthesized 79-bp region of the COCH gene of zebrafish (*Danio rerio*) (VBC, Genomics, Vienna, Austria) were included in a 25-μl real-time PCR reaction specific for that target. Obtained copy numbers were compared for reactions with or without the addition of isolated DNA from raw milk. A reduction in the obtained copy number or a negative result would indicate the presence of PCR inhibitors in the milk-derived DNA added to the PCR reaction.

Primers and probes (MWG Biotech, Ebersberg, Germany) for MAP-specific regions Mptb52.1, Mptb52.16 and Mptb54.33 were designed using the Primer Express^® ^Software v2.0 (Applied Biosystems, Foster City, CA, USA) (Table 2) [14]. These regions code for hypothetical proteins, with no functions assigned so far. Putative amplicons were checked for secondary structure formation using the mfold web server [18]. The 25-μl volume of the optimized PCR reaction targeting a 101-bp fragment of the Mptb52.16 region contained 20 mM Tris-HCl, 50 mM KCl, 3 mM MgCl_2_, 500 nM of each primer, 400 nM of probe, 200 μM (each) of dATP, dTTP, dGTP, and dCTP, 1.5 U of Platinum^® ^*Taq *DNA polymerase (Invitrogen, Carlsbad, CA, USA) and 6 μl DNA suspension. In addition, each reaction included 50 copies of an internal amplification control (IAC) based on the cytochrome oxidase subunit 3 gene (*co3*) from *Boa constrictor *to exclude false negative results [19]. IAC-specific primers and probe were added at concentrations of 200 nM each and are also listed in Table 2. Amplification in an MX3000p real-time PCR thermocycler (Stratagene, La Jolla, CA, USA) after initial denaturation at 94°C for 2 min was performed in 50 cycles, at 94°C for 20 sec, and 64°C for 1 min. Real-time PCR-derived copy numbers of the target region were expressed as MAP-specific bacterial cell equivalents (bce).

**Table 2:** Primers and probes used for real-time PCR

Target	Oligonucleotide	Function
Mptb52.1	5'-GCT CGC CGT GAT GTT GTT G-3'	forward primer
	5'-FAM-CTT GAC TCA GAT GCG GTG GAT GGA-BHQ_1_-3'	probe
	5'-CCC GAA AGC CCT TCT CAA G-3'rr	reverse primer
Mptb52.16	5'-CGA CAC CCC TCC AAT TGA TC-3'	forward primer
	5'-FAM-TTC CGC ACC CCT GAT GGA GTG T-BHQ_1_-3'	probe
	5'-ACC CGG AAG ATT GTC ACC G-3'	reverse primer
Mptb54.33	5'-CTC CTT CCA CGT CAG AAG CC-3'	forward primer
	5'-FAM-TTA CCA GTC ATC GGA GCC AGG TCG-BHQ_1_-3'	probe
	5'-GGA CGA CAC CAC TTG AAG AGC-3'	reverse primer
IAC	5'-TCA CAG CCC TCC AAC TAT CAG AA-3'	forward primer
	5'-HEX-TTC GTA GCC ACT GGG TTC CAC GG-BHQ_1_-3'	probe
	5'-TGT TGT CCC AAT CAT CAC GTG TA-3'	reverse primer

### Real-time RT-PCR

A 5-μl volume of RNA was reverse-transcribed into complementary DNA (cDNA) using 2 pmol Mptb52.16 specific primers and SuperScript III reverse transcriptase (Invitrogen) according to the manufacturer's instructions. RNase OUT recombinant RNase inhibitor (Invitrogen) was included in the cDNA synthesis reaction. Five microliter aliquots of the 20-μl volume of cDNA template generated were used for real-time PCR analysis as described above. The RT- control included 1.2 μl of un-transcribed RNA, which equals the quantity of RNA in the 5-μl aliquot of cDNA.

## Results and Discussion

### Development and optimization of the real-time PCR assay

The objective of the present study was to develop a real-time PCR assay for detection and quantification of MAP in milk, with the additional option of differentiating between viable and dead cells. The gene fragments Mptb52.1, Mptb52.16, Mptb52.33 were selected for the analysis because they are specific for MAP and were shown to be expressed in pure cultures in Dubos broth [14]. Primer and TaqMan^® ^probes were designed and each assay was tested with DNA isolated from raw milk (Table 2).

During optimization of the assays, severe interference of the raw milk background (e.g. DNA from microflora as well as DNA from somatic cells) was noted with all three assays when performing agarose gel electrophoresis of the amplicons (data not shown). As these problems were not grossly evident on the real-time PCR amplification curves, but competition for reagents during formation of unspecific amplicons might be detrimental for the detection of low contamination levels, these findings confirm the necessity to perform gel analysis when optimizing real-time PCR assays for environmental samples. Only the Mptb52.16 target could be optimized by increasing the combined annealing/extension temperature and by decreasing the MgCl_2 _concentration without sacrificing the efficiency of the real-time PCR reaction. BLAST search indicated that the selected target sequences for Mbtp52.16 specific primers and probes display 100% identity with MAP strains only. Partial identity with different species of other bacteria for each of the oligonucleotides was noted but regarded as not sufficient to enable detection with the selected primers/probe combination. However, it might have been the reason for the observed interference of the raw milk background revealed by agarose gel electrophoresis.

**Figure 1:**
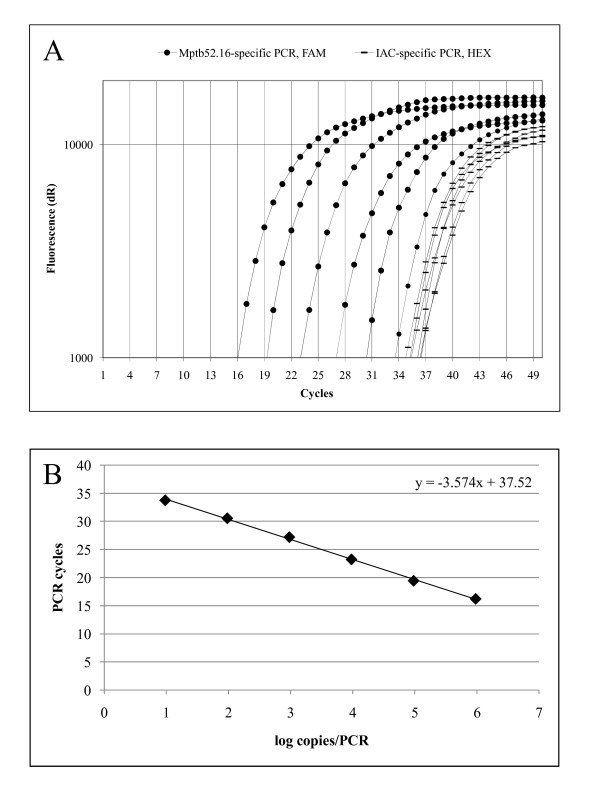
**Amplification plot (A) and standard curve (B) of the optimized real-time PCR assay for the Mptb52.16 target**. Based on the molecular weight of the genome of MAP strain K-10 (GenBank accession number AE016958), 1 ng DNA equals 9.59 × 10^5 ^copies of the entire genome. The MAP-specific target Mptb52.16 is a single-copy gene. Thus, this figure equals the number of PCR targets per ng. The dilution series ranged from 9.59 × 10^5 ^to 9.59 × 10^0 ^copies of the genomic DNA from MAP strain CIP 103974 per PCR. Fifty copies of an IAC were added to each reaction. The MAP-specific probe was detected in the FAM channel, whereas the IAC-specific probe was detected in the HEX channel.

The addition of 50 copies of an IAC to the optimized assay permitted identification of false negative results without exerting a negative effect on the MAP-specific real-time PCR (Figure 1). The amplification efficiency of the real-time PCR reaction with and without the addition of the IAC was 90.5% and 87.2%, respectively.

Since contamination levels in raw milk are low (e.g. l00 cfu/ml in milk from symptomatic and 2-8 cfu/50 ml in milk from asymptomatic cows) [4] and bacteria are not homogenously distributed at low contamination levels, it was decided to pool and concentrate the DNA isolated from four 15-ml aliquots to a final volume of 28 μl. Since 6 μl were transferred to the PCR reaction, a volume of 12.8 ml milk could be analyzed in a single PCR reaction. Performing four PCR reactions per sample permitted to screen a volume of 51 ml milk. It was confirmed that this approach did not lead to the concentration of PCR inhibitors (data not shown). In addition, when analyzing field samples, no false negative results were indicated by the integrated IAC.

Inclusivity and exclusivity testing of pure cultures of MAP (n = 10), non-MAP mycobacteria (n = 22) and raw milk microflora (n = 16) yielded no false positive or false negative results (Table 1), thus confirmed the specificity of the selected target, which was reported to be a drawback of the widely used IS900 target because other mycobacteria harboring similar sequences were observed [11-13]. Exclusivity testing could be expanded further by including members of the *Mycobacterium tuberculosis *complex and mycobacteria other than *M. xenopii*, harboring IS900-like elements such as *M. chelonae*, *M. terrae*, and *M. porcinum *[13,20]. However, since the selected target is different to IS900, specificity issues with those might not be expected.

### Detection limit in artificially contaminated raw milk samples

When determining the detection limit, great care was taken to avoid the introduction of large quantities of free DNA into the artificially contaminated milk samples, which might cause a bias towards a lower detection limit determined with a DNA-based method. To avoid overestimation, the bacterial suspension was washed thoroughly [21]. Cells were counted with a microscope because growth-related counts tend to underestimate the number of MAP cells present in a bacterial suspension [4,22]. Differences ranging up to 2 log scales were observed when counting cells in a counting chamber compared to performing colony counts [21].

Testing different batches of raw milk as well as pasteurized milk and UHT milk to confirm the absence of the MAP-specific target in the milk for artificial contamination experiments revealed a low level of contamination in some of the tested samples. Of two pasteurized milk samples and one UHT milk sample, one of the first and the latter yielded positive results. The contamination level was low: 8.7 × 10^-1 ^bce/ml pasteurized milk and 2.7 × 10^0 ^bce/ml UHT milk. A conventional commercially available PCR assay based on an alternative target (IS*Mav2*) was used to verify the presence of MAP DNA in these samples, and yielded identical results. As all process controls were negative, we could rule out laboratory contamination. Other authors have also observed low numbers of PCR-positive and occasionally even culture-positive pasteurized milk samples [16,23,24]. Eventually, a consistently negative batch of raw milk was identified and used for further experiments.

A dilution series of MAP cells in milk containing 2.42 × 10^5 ^to 2.42 × 10^-1 ^MAP cells/ml milk was prepared and analyzed with the real-time PCR assay. The obtained detection limit (2.42 × 10^1 ^MAP cells/ml milk) was comparable to the results achieved with other real-time PCR assays [22,25-29]. The majority of the published DNA isolation methods were based on mechanical cell lysis. For the present study we used a non-mechanical method of cell lysis which was successfully applied for real-time PCR-based detection of the Gram-positive bacterial species *Listeria monocytogenes *in milk [15]. This protocol includes multiple incubation and washing steps at 45°C in combination with solvents and detergents, and maybe permits the recovery of MAP from milk fat as well [25].

### Naturally contaminated milk samples

Analysis of 46 pre-milk and 52 main milk samples obtained from farms with a known history of Johne's disease yielded 47.8% MAP-positive pre-milk and 51.9% MAP-positive main milk samples, which is somewhat higher than the data reported by other authors. These farms had cows with a positive blood ELISA, fecal culture, or both, and were participating in a MAP-control program. Twenty-five paired pre-milk and main milk samples were available. Nine of these (36%) were positive in pre-milk only, eight (32%) in main milk only, and one sample (4%) was positive in both types of milk. Some authors observed 32.5% PCR-positive pre-milk samples in a farm with two cows shedding MAP [27], whereas others observed 33% PCR-positive main milk samples under similar circumstances [30]. Lower numbers are usually reported for farms with no cases of diarrhea or weight loss [31]. Comparisons of data are rendered difficult by the fact that different methods were used for DNA isolation and different volumes of milk were analyzed. We analyzed the DNA isolated from 51 ml of milk per sample, whereas other authors had performed PCR analysis on the DNA of 0.1 to 2.5 ml milk [4,22,25-27]. Contamination of the pre-milk and the main milk samples ranged from 1 × 10^0 ^to 5.1 × 10^2 ^bce/51 ml and from 1 × 10^0 ^to 6.5 × 10^1 ^bce/51 ml milk, respectively, with 90.9% of positive pre-milk and 96.3% of positive main milk samples having less than one bce per ml milk. Almost no quantitative data were available for comparison. A few tens to no more than 560 MAP cells/ml milk were detected by real-time PCR in individual milk samples of a herd with a known history of paratuberculosis [27]. Other authors reported less than 100 MAP cells/ml in bulk tank milk, including herds which were MAP positive by environmental culture [25].

### Exploring the potential for detection of viable MAP

Detection of RNA might indicate the presence of metabolically active MAP in the sample and could be performed either directly in the sample or after a short incubation period in broth. Transcription of the Mptb52.16 target was analyzed in artificially contaminated 15-ml volumes of raw milk and in 5-ml volumes of Dubos broth inoculated with bacteria collected from the 15-ml volumes of milk. The contamination level of raw milk was 5.1 × 10^6 ^MAP cells/15 ml. From this sample, 2.2 × 10^6 ^DNA targets and 1.0 × 10^4 ^RNA targets were recovered. After one week of incubation in Dubos broth, 8.8 × 10^5 ^DNA and 1.3 × 10^4 ^RNA targets were identified. The numbers of DNA and RNA targets obtained after an additional week of incubation in Dubos broth was similar (1.0 × 10^6 ^DNA and 9.6 × 10^3 ^RNA targets). These data suggest that there was no detectable growth of MAP or increase in the expression of the Mptb52.16-specific target during the observation period. The difference in RNA and DNA target number could indicate a low level of expression of the selected target or low metabolic activity of the MAP cells in general, which could be characterized further at the 16 S rRNA level [32]. On the other hand, the efficiency of RNA isolation might differ from the efficiency of DNA isolation or free DNA could still be transferred into the sample together with the bacterial cells. In addition, insufficient reverse transcription efficiency might have influenced the result. The RT- control indicated the presence of 7.5% DNA in RNA isolated from raw milk. After one week of incubation in Dubos broth, only one of two replicates of the RT- control remained positive on real-time PCR. After the additional week of incubation, no positive signal was identified in the RT- control.

## Conclusion

Concentrating the DNA of a large sample volume in combination with the newly developed real-time PCR assay permitted quantification of low levels of MAP cells in raw milk and pasteurized milk. The selected target - Mptb52.16 - is promising with regard to the detection of viable MAP and warrants further exploration. Given the low number of MAP targets detected in naturally contaminated samples, RNA based detection of viable MAP cells still provides a challenge. Future studies integrating quantitative DNA-based and RNA data as well as culture data might provide important information for risk assessment concerning the presence of MAP in raw milk and pasteurized milk.

## Abbreviations

bce: bacterial cell equivalents; cDNA: complementary deoxyribonucleic acid; CFU: colony forming units; MAP: *Mycobacterium avium *subsp. *paratuberculosis*; PCR: polymerase chain reaction; RT- control: minus reverse transcriptase control.

## Competing interests

The authors declare that they have no competing interests.

## Authors' contributions

MD contributed to carrying out the DNA isolation, carried out the RNA isolation and real-time PCR, and contributed to the analysis of data and writing of the manuscript. PV collected the field samples and contributed to carrying out the DNA isolation. JK and WB contributed to the conception and design of the study. MW contributed to the conception and design of the study and provided the research facilities. IH contributed to the conception and design of the study, data analysis, and drafting and writing of the manuscript. All authors have read and approved the final manuscript.
